# Deubiquitination of FANCD2 Is Required for DNA Crosslink Repair

**DOI:** 10.1016/j.molcel.2007.09.020

**Published:** 2007-12-14

**Authors:** Vibe H. Oestergaard, Frederic Langevin, Hendrik J. Kuiken, Paul Pace, Wojciech Niedzwiedz, Laura J. Simpson, Mioko Ohzeki, Minoru Takata, Julian E. Sale, Ketan J. Patel

**Affiliations:** 1 Medical Research Council, Laboratory of Molecular Biology, Hills Road, Cambridge CB2 0QH, UK; 2 Department of Medicine, Addenbrookes Hospital, Hills Road, Cambridge CB2 2QQ, UK; 3 Department of Human Genetics, Research Institute for Radiation Biology and Medicine, Hiroshima University,1-2-3 Kasumi, Minami-ku, Hiroshima, Japan 734-8553

**Keywords:** DNA, PROTEINS

## Abstract

Monoubiquitination of FANCD2 and PCNA promotes DNA repair. It causes chromatin accumulation of FANCD2 and facilitates PCNA's recruitment of translesion polymerases to stalled replication. USP1, a protease that removes monoubiquitin from FANCD2 and PCNA, was thought to reverse the DNA damage response of these substrates. We disrupted *USP1* in chicken cells to dissect its role in a stable genetic system. *USP1* ablation increases FANCD2 and PCNA monoubiquitination but unexpectedly results in DNA crosslinker sensitivity. This defective DNA repair is associated with constitutively chromatin-bound, monoubiquitinated FANCD2. In contrast, persistent PCNA monoubiquitination has negligible impact on DNA repair or mutagenesis. USP1 was previously shown to autocleave after DNA damage. In DT40, USP1 autocleavage is not stimulated by DNA damage, and expressing a noncleavable mutant in the *USP1* knockout strain partially rescues crosslinker sensitivity. We conclude that efficient DNA crosslink repair requires FANCD2 deubiquitination, whereas FANCD2 monoubiquitination is not dependent on USP1 autocleavage.

## Introduction

The ability to target DNA repair enzymes to specific sites in the genome is essential for proper and appropriate DNA repair. Once repair is completed, the repair factors have to be inactivated to avert inappropriate action and corruption of genetic information. A means by which this regulation is achieved is by the posttranslational modification of repair proteins, and it is now clear that interconnected signal transduction pathways orchestrate such modifications (Zhou and Elledge, 2000). Recently, much focus has been placed on ubiquitination as an important modification. This modification not only targets proteins for degradation but can also determine subcellular localization and promote protein-protein interactions (Huang and D'Andrea, 2006). Although much progress has been made in determining how ubiquitination is achieved, the importance of the reversal of this modification has only recently been appreciated. Removal of both polyubiquitin and monoubiquitin from target proteins is dependent on a specific class of enzymes, the ubiquitin hydrolases (Nijman et al., 2005b).

The USP1 protein is a ubiquitin hydrolase that removes monoubiquitin from the Fanconi anemia D2 protein (FANCD2) (Nijman et al., 2005a) and from the proliferating cell nuclear antigen (PCNA) (Huang et al., 2006). The monoubiquitination of FANCD2 by the FA nuclear complex is a crucial step in the activation of the Fanconi anemia DNA repair pathway (Garcia-Higuera et al., 2001). Mutational inactivation of any one of the proteins that are part of the FA core complex results in loss of FANCD2 monoubiquitination and as a result the loss of function of this repair pathway (Garcia-Higuera et al., 2001). Monoubiquitination targets FANCD2 to chromatin at sites of DNA damage (Montes de Oca et al., 2005), and *USP1* knockdown in human cells results in an accumulation of monoubiquitinated FANCD2. There is some evidence that this leads to constitutive DNA repair activity (Nijman et al., 2005a). In addition to the persistence of FANCD2 monoubiquitination, *USP1* knockdown also results in an increased level of steady-state PCNA monoubiquitination (Huang et al., 2006). The monoubiquitination of PCNA is a highly conserved response in eukaryotes. When DNA damage impedes replication, RAD18 and RAD6 form an active ubiquitin-conjugating complex that transfers a single ubiquitin to K164 of PCNA (Hoege et al., 2002). This modification facilitates the recruitment of a special class of translesion DNA polymerases to PCNA to promote replication across damaged DNA (Bienko et al., 2005; Kannouche et al., 2004). Studies using small interfering RNA (siRNA) against *USP1* in human cell lines suggest that persistent PCNA monoubiquitination leads to the spontaneous localization of translesion polymerases to nuclear foci (Huang et al., 2006). In addition, this study reported elevated levels of mutagenesis in ultraviolet (UV)-light-damaged plasmids that were transiently introduced into such cells. The current evidence therefore points to the deubiquitination of both FANCD2 and PCNA as being important in the regulation of their function for the DNA damage response.

Because studies in human cells point toward an important role for USP1 in the regulation of repair by PCNA and FANCD2, the regulation of USP1 itself has become important. In particular, how do USP1 levels and activity correlate with the modification of these two substrates? Both modifications are induced during DNA replication (Taniguchi et al., 2002) but are potentiated further by DNA damage. Indeed, USP1 messenger RNA (mRNA) appears to be under cell-cycle control, peaking during the S phase and diminishing during the G1 phase (Nijman et al., 2005a). Furthermore, the USP1 protein itself is reported to undergo rapid degradation in human cells after UV-induced DNA damage (Huang et al., 2006). This degradation appears to require an intrinsic activity of USP1 that cleaves a glycine-glycine linker sequence within its own polypeptide. Thus, USP1 autodegradation upon DNA damage is postulated to be a mechanism behind DNA damage-inducible FANCD2 and PCNA monoubiquitination (Friedberg, 2006). The striking mechanism by which USP1 inactivates itself has interesting parallels with the bacterial LexA system (Ulrich, 2006). Here, the autoproteolysis of the LexA protein, stimulated by RecA, after DNA damage results in the activation of DNA repair. It is therefore of considerable importance to determine the mechanism of USP1 regulation, particularly how it is possible for this protein to sense DNA damage and relay such a signal to catalyze autodegradation.

In this study, we created a DT40 knockout of *USP1* to address the role of this protein in DNA repair in a stable genetic system. We have used this to clarify whether USP1-mediated deubiquitination functions to switch off DNA repair and how specific and crucial self-destruction is for the regulation by this enzyme. The studies presented here lead us to reappraise the role of USP1 in DNA repair and mutagenesis.

## Results

### FANCD2 and PCNA Are Substrates for Chicken USP1

**Figure 1:**
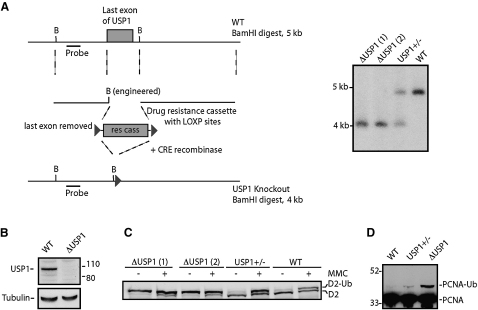
Disruption of the Chicken *USP1* Gene Results in Accumulation of Monoubiquitinated FANCD2 and PCNA (A) Map of the *USP1* locus and design of gene disruption cassette. The large terminal exon, which is removed by homologous integration, contains residues that are part of the active site. Genomic DNA digested with BamHI (B) gives a 5 kb band for wild-type DT40 (WT) and 4 kb band for the *USP1* knockout. (B) USP1 western blot of extract derived from WT and Δ*USP1* cells. (C) FANCD2 western blot of extract derived from the WT, *USP1^+/−^*, and two clones of Δ*USP1*. Whole-cell extracts were prepared from the indicated cell line before (−) or after (+) exposure to MMC. (D) PCNA western blot on extract derived from untreated WT, *USP1^+/−^*, or Δ*USP1* cells.

In order to establish a genetic system to study the function of USP1, we disrupted the *USP1* locus in the avian B cell line DT40. The chicken *USP1* gene was cloned, and its locus was identified and mapped. The last exon of the *USP1* gene encodes a histidine motif that is essential for catalytic activity (Nijman et al., 2005a). This information was used to design a gene disruption construct, which deletes this large exon. Both alleles were disrupted by sequential transfection, and the disruption was confirmed by Southern analysis (Figure 1A). To confirm at the protein level that the *USP1* gene had been disrupted, a western blot was performed with an antibody directed against the N terminus of human USP1. As can be seen from Figure 1B, a band of approximately 105 kDa was present in the whole-cell lysate of the wild-type DT40 cells but absent in the *USP1* knockout cell line. The *USP1* knockout cell line will be referred to as Δ*USP1*. siRNA studies in human cells have indicated that both PCNA (Huang et al., 2006) and FANCD2 (Nijman et al., 2005a) are substrates for the deubiquitination activity of USP1. To confirm these findings, we analyzed the levels of monoubiquitinated FANCD2 and PCNA in the Δ*USP1* strain by western blot. As seen from the western analysis (Figure 1C), FANCD2 was predominantly present in its monoubiquitinated form in Δ*USP1* irrespective of DNA damage. Similarly, a western blot for PCNA (Figure 1D) confirms that the level of monoubiquitinated PCNA is substantially increased in the Δ*USP1* strain when compared to the wild-type control (Simpson et al., 2006). Therefore, the disruption of the *USP1* gene in DT40 confirms that FANCD2 and PCNA are substrates for this enzyme and faithfully recapitulates the siRNA knockdown studies performed in human cells. This is not surprising, given the high level of identity between chicken and human USP1. An alignment of the two proteins is shown in Figure S1 the Supplemental Data available with this article online.

### Accumulation of Ubiquitinated PCNA Does Not Stimulate Mutagenic Repair in the Ig Locus

PCNA monoubiquitination has been shown to promote mutagenic DNA damage bypass and repair. This is because monoubiquitinated PCNA is capable of binding and recruiting the translesion polymerases to sites of DNA damage by interacting with their UBM and UBZ ubiquitin-binding motifs (Bienko et al., 2005). The available evidence on the effect of persistent PCNA ubiquitination on mutagenesis suggests that it results in additional recruitment of mutagenic bypass. DT40 cells allow us to test whether such a potentiation of mutagenesis is observed during the bypass of endogenously generated genomic lesions. The immunoglobulin (Ig) loci of DT40 are continuously diversified by both gene conversion from upstream donor pseudogenes and nontemplated point mutation. This diversification is initiated by abasic sites formed by the concerted action of activation-induced deaminase (AID) and uracil DNA glycosylase (UNG) (Di Noia and Neuberger, 2002; Di Noia et al., 2006). Abasic-site-induced gene conversion is dependent on homologous recombination (Sale et al., 2001) and has been reported to also require DNA polymerase η (Kawamoto et al., 2005), a translesion polymerase recruited by PCNA ubiquitination (Kannouche et al., 2004). Point mutation is dependent on mutagenic bypass of the abasic site and involves at least REV1 and the monoubiquitination of PCNA (Simpson and Sale, 2003; Arakawa et al., 2006). We therefore asked whether persistent monoubiquitinated PCNA impacted either of these processes.

**Figure 2:**
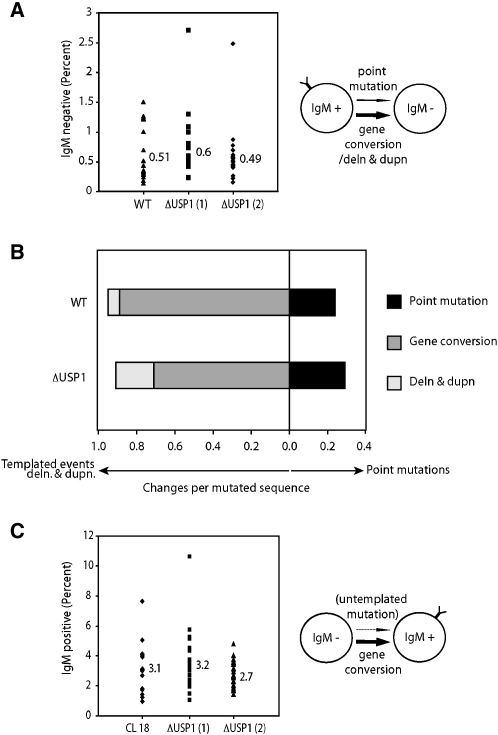
Normal Templated and Untemplated Diversification of the Ig Locus in Δ*USP1* Cells (A) Loss of surface IgM fluctuation analysis in the WT and two clones of Δ*USP1* strains. For each cell line, 24 clones were individually expanded for up to 30 days, after which they were assayed by FACS for loss of sIgM expression. Numbers depict the median value for the prevalence of sIgM-negative cells. (B) Quantitation of mutational events in sorted sIgM-loss variants. Mutations were expressed as changes per altered sequence that were templated (gene conversion), deletions, or duplications and point mutations (untemplated). In total, 45 different mutated sequences (where clonality was eliminated) were compared to a large existing WT database of 456 sequences. (C) Gain of sIgM fluctuation analysis of the clone 18 frameshift. In this cell line, gene conversion will restore sIgM expression. Twenty-four clones from the WT (CL 18) and two clones of Δ*USP1* were expanded in culture for 30 days. Surface IgM was determined by FACS analysis, and the indicated number denotes the median value for the prevalence of sIgM expression.

The overall diversification in the immunoglobulin loci of DT40 can be assessed by monitoring the loss of surface immunoglobulin (sIgM) expression in a fluctuation analysis starting from multiple sIgM-positive subclones. Single sIgM-positive cells were sorted and expanded for 4 weeks, after which the proportion of sIgM-negative cells was assessed by flow cytometry (Figure 2A). Wild-type clones generated a median of 0.51% sIgM-loss variants, and two clones of Δ*USP1* generated 0.60% and 0.49% respectively. To examine whether there was any difference in the pattern of diversification, we sequenced the rearranged Ig light chain variable region (VL) from Δ*USP1* sIgM-loss variants. This revealed an average of 0.71 gene conversions per mutated sequence and 0.29 nontemplated point mutations in Δ*USP1* (Figure 2B). These proportions are directly comparable to data obtained from a large database of VL sequences from wild-type DT40 cells that were collected under identical conditions (Ross and Sale, 2006) (0.89 for gene conversions and 0.24 for point mutations). A small increase in the number of deletions and duplications was seen by sequencing. In order to exclude an impairment of Ig gene conversion, we carried out an assay for the reversion of sIgM negative in the CL 18 line (Buerstedde et al., 1990). This clone contains an inactivating frameshift mutation in the IgV, which can be rescued by a gene conversion (Figure 2C). The gain of sIgM expression in 24 subclones of wild-type or Δ*USP1* expanded for 4 weeks was assayed by flow cytometry. Wild-type cells generated a median of 3.1% sIgM-positive cells, whereas Δ*USP1* generated 3.2% and 2.7%, indicating that gene conversion frequency is not affected. A very clear conclusion is that the level of point mutations occurring in this locus is not influenced by the loss of USP1. In summary, elevated levels of monoubiquitinated PCNA are not sufficient to increase mutagenesis at endogenously created DNA damage in the Ig locus.

### The Δ*USP1* Strain Is Hypersensitive to DNA Crosslinking Agents

**Figure 3:**
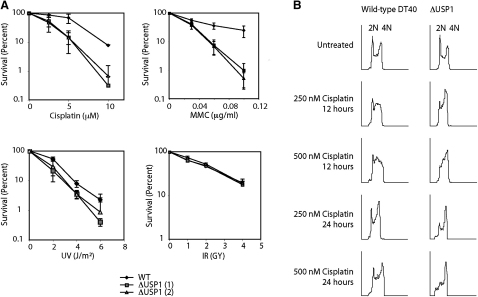
USP1 Contributes to DNA Damage Tolerance (A) Cellular sensitivity to DNA-damaging agents as determined by colony survival assay. The top left shows cisplatin, and the top right shows mitomycin C (MMC). The bottom left shows UV, and the bottom right shows X-rays. Error bars represent one standard deviation (SD). (B) Propidium iodide cell-cycle analysis of wild-type DT40 and Δ*USP1* after exposure to different doses of cisplatin for 12 or 24 hr. 2N represents the G1 peak and 4N represents the G2/M peak.

siRNA knockdown of *USP1* in human cells appears to be well tolerated and confers cellular resistance to DNA damage (Nijman et al., 2005a). This moderate effect could be due to a constitutive induction of DNA repair. Direct extrapolation from these studies would predict that the Δ*USP1* strain should also be resistant to DNA damage. We therefore tested, by colony survival assay, the response of the Δ*USP1* strain to the DNA crosslinking agents cisplatin and mitomycin C (MMC), as well as UV irradiation and X-rays. The data in Figure 3A clearly show that Δ*USP1* is hypersensitive to crosslinks (LD10 for Δ*USP1* is 5.5 μM cisplatin; LD10 for wild-type is 9.5 μM cisplatin) and marginally sensitive to UV light but not sensitive to X-rays. Cell-cycle analyses after exposure to cisplatin reveal a corresponding cell-cycle response to DNA damage by the Δ*USP1* cell line (Figure 3B). In summary, these results unexpectedly reveal that the inactivation of the *USP1* gene results in DNA crosslinker hypersensitivity.

### Persistent Monoubiquitinated FANCD2 Is Responsible for DNA Crosslinker Sensitivity

**Figure 4:**
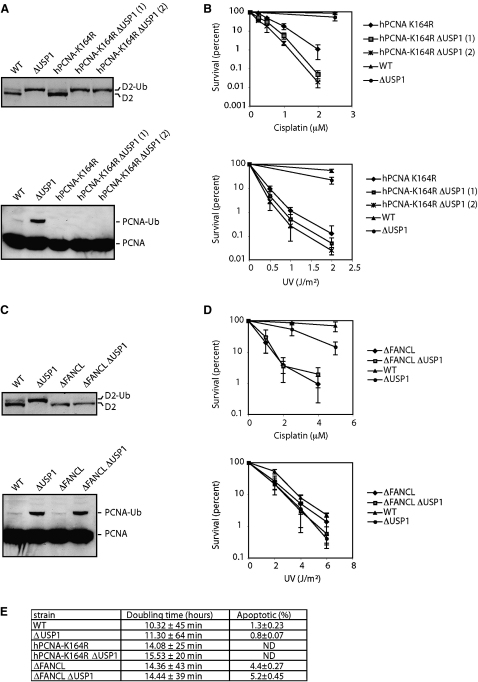
Persistent Monoubiquitinated FANCD2 and Not PCNA Is Responsible for DNA Damage Sensitivity (A) FANCD2 (top) and PCNA (bottom) western blot of WT, Δ*USP1*, hPCNA-K164R knockin, and hPCNA-K164R Δ*USP1* cell extracts. (B) Colony survival assay with the same strains exposed to low doses of cisplatin or UV light. Error bars represent one SD. (C) FANCD2 (top) and PCNA (bottom) western blot of WT, Δ*USP1*, Δ*FANCL*, and Δ*FANCL*Δ*USP1* cell extracts. (D) Colony survival assay with the same strains exposed to low doses of cisplatin (top) and high doses of UV light (bottom). Error bars represent one SD. (E) Table showing doubling times and the prevalence of apoptotic cells in the strains tested in this section.

PCNA and FANCD2 are the two known substrates of USP1. We therefore wished to determine whether the persistent monoubiquitination of either of these substrates was responsible for corrupting DNA repair. To determine whether crosslinker hypersensitivity is due to persistent PCNA modification, we disrupted *USP1* in a DT40 strain in which the endogenous PCNA locus has been disrupted and the cells rescued by ectopic expression of a point mutated version of human PCNA that cannot be monoubiquitinated (PCNA-K164R). This cell line had previously been created and characterised by Simpson et al. (2006). The ablation of *USP1* in this strain shows very clearly that PCNA monoubiquitination is undetectable, whereas modified FANCD2 persists (Figure 4A). The PCNA-K164R strain is hypersensitive to both UV light and cisplatin; with both kinds of DNA damage, this is more marked than in the Δ*USP1* single-knockout strain. We therefore exposed the relevant single and double mutants to very low doses of UV light and cisplatin. As can be seen in Figure 4B, *USP1* disruption has an additive impact on cisplatin sensitivity (LD10 for hPCNA-K164R Δ*USP1* is 0.74 μM; LD10 for hPCNA-K164R is 1.21 μM), whereas the effect on UV sensitivity is marginal. To determine the contribution of persistently ubiquitinated FANCD2 to the observed crosslinker sensitivity in Δ*USP1*, we disrupted *USP1* in a Δ*FANCL* strain. FANCL is the ubiquitin ligase component of the FA core complex (Meetei et al., 2005), so its disruption leads to a complete block in FANCD2 but not PCNA modification (Figure 4C). The sensitivity of the Δ*FANCL* strain to crosslinks is more marked than that of Δ*USP1*. The double Δ*FANCL*Δ*USP1* is just as sensitive to cisplatin as the single Δ*FANCL* knockout strain (Figure 4D).

Furthermore, as seen in Figure 4E, disruption of the *USP1* gene in the Δ*FANCL* cell line does not have a negative impact on growth rate, further demonstrating the epistatic relationship between *FANCL* and *USP1*. Taken together, this genetic analysis therefore indicates that persistent FANCD2 ubiquitination and not PCNA or any unknown substrate is responsible for the DNA crosslinker sensitivity in the Δ*USP1* strain.

### USP1 Regulates FANCD2 Accumulation on Chromatin

**Figure 5:**
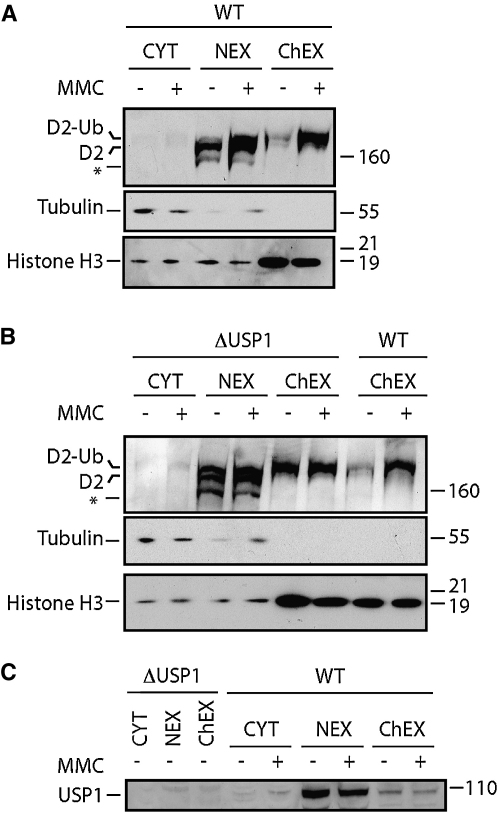
Lack of USP1-Mediated FANCD2 Deubiquitination Results in the Accumulation of FANCD2 on Chromatin (A and B) Subcellular fractionations of the wild-type (A) and Δ*USP1* (B) cell lines before (−) and after (+) DNA damage (150 ng/ml MMC for 14 hr). The following abbreviations are used: cytoplasmic fraction (CYT), nuclear extracts (NEX), and solubilized chromatin fraction (ChEX). Filters were probed with antibodies to FANCD2, tubulin, or histone H3. The asterisk denotes an unspecific band. (C) Samples from the same subcellular fractionation assays were used for western blot against USP1. The samples from the untreated Δ*USP1* cell line were used as a negative control.

So far, we have presented strong genetic evidence that the *USP1* gene functions as a positive regulator of DNA repair and that FANCD2 is physiologically its most important known target in the DNA repair response. We therefore wished to establish how USP1 facilitates the function of FANCD2. Currently, it is unclear whether FANCD2 has an enzymatic function, although it is known that it is recruited onto chromatin after DNA damage (Montes de Oca et al., 2005). Indeed, as shown in Figure 5A, DT40 cells display a striking accumulation of FANCD2 in the chromatin fraction upon exposure to the DNA crosslinker mitomycin C. This response is abnormal in the Δ*USP1* strain. Figure 5B clearly reveals that even in the absence of DNA damage, monoubiquitinated FANCD2 is constitutively present in the chromatin fraction. In conclusion, a function of USP1 is to ensure that the accumulation of FANCD2 on chromatin is temporally restricted to occurring after DNA damage. In addition, we investigated the localization of USP1 before and after the induction of DNA crosslinks. We find that USP1 is primarily localized in the nuclear extract and that, unlike FANCD2, it does not change localization after DNA damage (Figure 5C).

### Degradation of Chicken USP1 Coincides with Apoptosis

**Figure 6:**
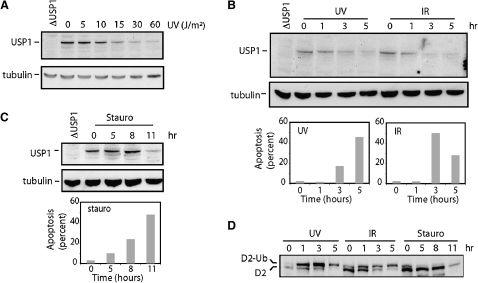
USP1 Degradation in Response to DNA Damage Correlates with Apoptosis (A) Western blot to detect USP1 3 hr after the exposure of cells to a range of different doses of UV light. A tubulin western blot was used as a loading control. (B) The top shows USP1 and tubulin western blots of extracts from cells exposed to UV light (30 J/m^2^) and X-rays (20 GY) recovered for 1, 3 and 5 hr. The bottom shows a column chart with the percentage of apoptotic cells as determined by FACS analysis of Annexin V uptake at the same time points. (C) The top shows USP1 and tubulin western blots of extracts from cells exposed to the non-DNA-damaging apoptosis-inducing drug staurosporine. Samples were removed at 5, 8 and 11 hr after addition of the drug. The bottom shows a column chart with the percentage of apoptotic cells detected by uptake of Annexin V at the same time points. (D) FANCD2 western blot performed on the samples described above (treated with UV, X-rays, or staurosporine).

The proposed mechanism by which the cellular level of USP1 is regulated is a particularly intriguing aspect of this protein. Recent work has suggested that the protein is able to cleave its own peptide backbone when cells are exposed to high doses of UV irradiation (Huang et al., 2006). This led to the suggestion that USP1 can respond to a DNA damage signal by autodegradation. To investigate this phenomenon, we treated DT40 cells with different doses of UV light and subsequently examined the level of the endogenous USP1 by western blot (Figure 6A). We observed the UV-induced degradation of USP1, but it occurred only after very high doses of UV light. The LD10 dose for UV on DT40 cells is 3.75 J/m^2^, but USP1 degradation seems to require 10 J/m^2^ or more. We therefore analyzed cell viability and apoptosis by annexin 5 uptake. As can be seen in Figure 6B, after UV treatment, there is a sharp rise in the number of apoptotic cells at the point of USP1 degradation. The same phenomenon and correlation can be seen when the cells are exposed to X-rays (Figure 6B). Because both treatments induce DNA damage, we sought to determine whether USP1 degradation can occur when cells undergo non-DNA-damage-inducible apoptosis. The drug staurosporine is a potent inducer of apoptosis in DT40 cells, but this agent does not directly damage DNA. Strikingly, exposure to staurosporine results in degradation of USP1, and once again we notice a correlation between the levels of USP1 and the prevalence of apoptotic cells (Figure 6C). Genotoxic damage results in increased levels of monoubiquitinated FANCD2, and these increased levels have in part been proposed to be due to USP1 degradation (Huang et al., 2006). However, damage-induced FANCD2 monoubiquitination is already present at 1 hr after treatment, whereas USP1 degradation occurs later. Furthermore, it is notable that staurosporine-treated cells show no accumulation of ubiquitinated FANCD2 despite diminished levels of USP1 (Figure 6D). The experiments shown in Figure 6 were furthermore repeated with a DT40 cell line in which both alleles of USP1 were tagged with haemagglutin (HA) (Figure S2). Cumulatively, these results suggest that there is a correlation between apoptosis and USP1 degradation, and they demonstrate that the DNA-damage-induced persistence of monoubiquitination of FANCD2 occurs before USP1 disappears.

### DNA-Damage-Induced Cleavage of USP1 Is Not Dependent on Its Own Catalytic Activity

**Figure 7:**
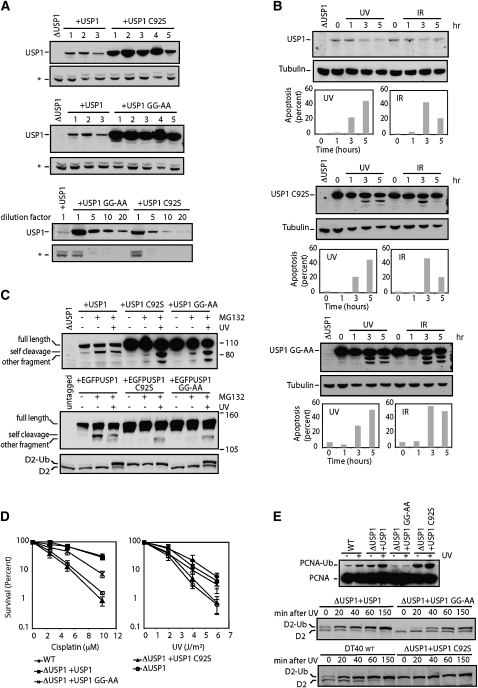
Mutation of Either the Glycine-Glycine Motif or the Active-Site Cysteine of USP1 Does Not Abolish DNA-Damage-Induced USP1 Cleavage (A) Δ*USP1* cells were transfected with cDNA encoding USP1 wild-type, USP1 GG-AA (glycine linker mutant), or USP1 C92S (active site mutant). The top and middle present western blots showing the level of USP1 in different clones stably transfected with either wild-type or mutant USP1 cDNA. The bottom shows serial dilution of extracts from clones expressing mutant USP1 compared to the WT USP1. A crossreacting band (^∗^) is shown as a loading control for the western blots. (B) Western blots to detect USP1 or tubulin in samples withdrawn at the indicated time points after exposure to UV or X-rays. At the same time points, samples were removed and assayed for the prevalence of apoptosis (lower panels). The top shows a Δ*USP1* strain stably transfected with wild-type *USP1* cDNA. The middle shows a Δ*USP1* strain stably transfected with active site mutant *USP1* cDNA (USP1 C92S). The bottom shows a Δ*USP1* strain stably transfected with *USP1* cDNA mutated in the cleavage motif (USP1 GG-AA). (C) The top shows a western blot for USP1 in the presence (+) or absence (−) of the proteasome inhibitor MG132 (10 μM for 3 hr) with (+) or without (−) 30 J/m^2^ of UV exposure (3 hr). The antibody recognizes the full-length USP1 (*full length*), the self cleavage fragment (*self cleavage*) and a non-self cleavage USP1 fragment (*other fragment*). The bottom shows a western blot for EGFP and FANCD2 in extracts from the Δ*USP1* strain stably transfected with N-terminally EGFP-fused *USP1* cDNA (wild-type and mutated). The EGFP antibody recognizes the full-length EGFP-USP1 (*full length*), the self cleavage fragment (*self cleavage*) and a non-self cleavage EGFP-USP1 fragment (*other fragment*). (D) Wild-type (*+USP1*) or mutant USP1 (*+USP1 C92S* or *+USP1 GG-AA*) cDNA stably transfected into the Δ*USP1* strain was tested for its ability to rescue the cisplatin or UV sensitivity by colony survival assay. Error bars represent one SD. (E) Cell lysates from the same respective cell lines were tested alongside each other with (+) or without (−) 30 J/m^2^ of UV exposure (150 min before harvest) so that the induction of PCNA monoubiquitination could be revealed (top). FANCD2 western blot of samples taken at different time points after UV irradiation (30 J/m^2^) is shown (bottom).

USP1 is thought to mediate its own destruction, cleaving at an internal glycine-glycine (Gly-Gly) linker sequence, leaving an N-terminal fragment, which is approximately 15 kDa smaller than the full length (Huang et al., 2006). To study the self cleavage of USP1, we stably transfected the Δ*USP1* strain with a plasmid, leading to the expression of wild-type USP1, a catalytic inactive form of USP1 (USP1 C92S), or USP1 mutated in the Gly-Gly motif (USP1 GG-AA). Strikingly, all clones obtained from transfections with either mutant USP1 isoform displayed very high levels of USP1 relative to those of clones transfected with the wild-type USP1 (Figure 7A), indicating that the two mutant USP1 forms were indeed incapable of self cleavage. Nevertheless, after exposure to both UV and X-rays, specific bands representing USP1 cleavage products appear below both mutant forms of USP1 (Figure 7B). This result shows that USP1 C92S and USP1 GG-AA, despite being incapable of self cleavage, still undergo DNA-damage-induced degradation, possibly due to apoptosis. The self cleavage product of USP1 is normally rapidly degraded by the proteasome. The proteasome inhibitor MG132 blocks this and enables the detection of the cleavage product. To address whether self cleavage contributes to the DNA-damage-induced degradation of the wild-type USP1, we added the proteasome inhibitor MG132 to UV-treated and untreated Δ*USP1* cells stably expressing wild-type or mutant USP1 (Figure 7C). As expected, the addition of proteasome inhibitor stabilized a USP1 fragment, which was specific for the wild-type USP1, demonstrating that this band corresponds to the self cleavage fragment. Strikingly, the level of this particular fragment was not increased after treatment with UV. To verify this finding, we repeated the same experiment with N-terminally enhanced green fluorescent protein (EGFP)-tagged USP1 proteins. Furthermore, we examined the level of FANCD2 ubiquitination in each sample to show that treatment with MG132 does not induce DNA damage. In summary, in DT40, the self cleavage of USP1 is important for maintaining appropriate levels of this protein. However, USP1 self cleavage is not increased by UV-induced DNA damage, which is certainly capable of inducing robust monoubiquitination of both FANCD2 and PCNA. In addition, we find that USP1 self cleavage was not regulated in a cell-cycle-specific manner because wild-type as well as the non-self cleavable USP1 mutants show no differences in levels at all phases of the cell cycle (Figure S3).

The hypersensitivity to cisplatin and UV can be complemented by the ectopic expression of wild-type USP1 (Figure 7D). We therefore asked whether the inactivation of self cleavage resulted in a significant functional impact on DNA repair capacity. Strikingly, the expression of USP1 GG-AA still largely rescues the cisplatin and UV sensitivity, despite the fact that this form was present at levels that were 20-fold higher than the wild-type protein. As expected, the catalytically inactive form of USP1 (USP1-C92S) did not complement.

Furthermore, the Δ*USP1* strain complemented with USP1 GG-AA can respond to DNA damage by the monoubiquitination of FANCD2, though with slightly slower kinetics (Figure 7E). This is probably due to the extremely high level of active USP1 and might explain the inability of USP1 GG-AA to fully complement the cisplatin sensitivity. The level of PCNA monoubiquitination was reproducibly lower in the Δ*USP1* strain complemented with USP1 GG-AA; however, this was not sufficient to result in any sensitivity to UV exposure (Figure 7D). In summary, the self cleavage of chicken USP1 is not induced by DNA damage and is not strictly required for USP1 to improve cellular DNA repair efficiency.

## Discussion

The studies presented here suggest an alternative view for the function of the USP1 protein in DNA damage response, namely that USP1 is a positive regulator of DNA repair. Its physiologically predominant known substrate is the FANCD2 protein, the deubiquitination of which functions to promote the shuttling of this protein onto chromatin. Although USP1 is capable of self cleavage, this process does not appear to be crucial for the regulation of its activity.

A surprising finding of this study is that accumulation of monoubiquitinated PCNA is well tolerated in Δ*USP1* cells. There appears to be a clear induction in mutagenic repair when UV-damaged plasmid substrates are recovered from human cells subjected to siRNA against USP1 (Huang et al., 2006). However, DT40 cells provide an opportunity to determine how the lack of USP1 affects mutagenic DNA damage bypass in the context of chromosomal DNA. Point mutations in the Ig variable regions of DT40 result from translesion synthesis across abasic sites generated by the concerted action of activation-induced deaminase and uracil DNA glycosylase. This response requires the Rev1 enzyme, as well as monoubiquitinated PCNA (Simpson and Sale, 2003; Arakawa et al., 2006). Therefore, a simple prediction would be that the prevalence of these mutations should be increased in the *USP1* knockout. But this is not what we see. Indeed, elevated PCNA monoubiquitination does not induce mutations, nor does it influence gene conversion events. The latter events are known to be facilitated by Polymerase η (Kawamoto et al., 2005), a translesion polymerase that is regulated by PCNA ubiquitination. In retrospect, it might not be surprising that persistent PCNA monoubiquitination has little impact because it is known that monoubiquitinated PCNA can still support normal replication in vitro (Garg and Burgers, 2005; Haracska et al., 2006). Therefore, PCNA modification might only recruit translesion polymerases at sites of stalled replication. If this is the case, then we would not expect elevated levels of monoubiquitinated PCNA to be sufficient to displace normal replication with translesion synthesis.

The previously published studies on USP1 indicated a role for deubiquitination in the downregulation of DNA repair. This stems from the observation that siRNA against *USP1* in human cells makes them slightly more resistant to DNA damage, prompting the explanation that persistently ubiquitinated FANCD2 or PCNA promote constitutive DNA repair. However, in stark contrast, we observed the opposite impact on DNA repair in the Δ*USP1* chicken DT40 cells. Although we do not know the reason for this difference, it is nevertheless most unlikely to reflect completely opposite functions for USP1 in different vertebrate species. The Fanconi anemia and the RAD6/RAD18 pathways are remarkably conserved both biochemically and functionally between humans and birds.

Our genetic dissection shows that the accumulation of monoubiquitinated FANCD2 and not PCNA is most likely to be responsible for the subversion of DNA repair. Because there is a constitutive accumulation of FANCD2 on chromatin in the absence of USP1, the most plausible reason for the observed DNA repair defect is the titration of FANCD2 away from damaged DNA into chromatin. In this instance, there would be a requirement for FANCD2 to be rapidly recycled from sites of repair. Alternatively, it might be very important to restrict FANCD2 monoubiquitination tightly to its function in repair; thus, for example, deubiquitination might facilitate the rapid removal of the protein from a site of ongoing repair. An important consideration is that the recently identified FANCI protein is a monoubiquitinated FANCD2-binding partner, and it seems that this protein is also a USP1 substrate (Smogorzewska et al., 2007). It will be interesting to determine how this protein is impacted by USP1. Many DNA repair transactions function in a sequential manner, and it might therefore be important to ensure that FANCD2 is removed to enable the next step to be initiated. Future studies will aim to determine whether any of these are the reason for the positive role USP1 plays in regulating the FA pathway.

As mentioned earlier, previous studies have hypothesized a remarkable mechanism by which USP1 is regulated. It was suggested that DNA-damage-inducible USP1 self cleavage sustains FANCD2 and PCNA monoubiquitination, thus promoting repair. Our studies test this hypothesis directly and demonstrate that this cannot be the predominant mode of regulation. First, it should be noted that in both the human and chicken systems, high doses of genotoxic agents are required for the degradation of USP1 (Huang et al., 2006; Ulrich, 2006). Here, we show that the degradation of USP1 coincides with the appearance of apoptotic cells. Moreover, there does not seem to be a strict correlation between the appearance of FANCD2 ubiquitination and USP1 degradation. The second issue that is investigated here concerns the auto cleavage of USP1 at a conserved Gly-Gly motif (Huang et al., 2006). It is clear that mutations in either the active site or at the Gly-Gly motif greatly stabilize USP1. Nevertheless, both of these mutants are cleaved after DNA-damage-induced apoptosis, whereas the self cleavage of the wild-type USP1 is not stimulated by UV-induced DNA damage. We therefore conclude that the apoptosis-induced fragmentation of USP1 does not involve self cleavage. It is also striking that despite being present at levels that are 10-fold to 20-fold higher than the wild-type protein, the functional impact of mutating the Gly-Gly motif in USP1 is actually minimal. Indeed, the kinetics of FANCD2 ubiquitination are marginally impacted, and this mutated form of USP1 largely complements the knockout cell line with respect to DNA crosslinker sensitivity.

In the future, it will be important to determine precisely how USP1 activity is regulated, whether it functions alone or requires another cellular cofactor. In summary, the studies presented here provide a potential framework for future experiments to define the role of USP1-dependent deubiquitination in the regulation of DNA repair.

## Experimental Procedures

### Gene Disruption in DT40

The chicken *USP1* locus was identified by BLASTing the human protein sequence against the ENSEMBL draft chicken genome sequence. Transfection, selection, and Southern analyses of targeted DT40 clones were carried out as previously described (Niedzwiedz et al., 2004).

### Cellular Subfractionations

Cellular subfractionations were performed as previously described (Mosedale et al., 2005), except that 3 mM N-ethylmaleimide (NEM) (Sigma) was included in the hypotonic buffer (10 mM Tris-HCl [pH 7.4], 10 mM KCl, 1.5 mM MgCl_2_, 10 mM 2-mercaptoethanol, 3 mM NEM, and protease inhibitor cocktail), as well as the high-salt buffer (15 mM Tris-HCl [pH 7.4], 1 mM ethylenediaminetetraacetic acid (EDTA), 500 mM NaCl, 1 mM MgCl_2_, 10% glycerol, 10 mM 2-mercaptoethanol, 3 mM NEM and protease inhibitor cocktail).

### Cell Doubling Time and Apoptosis

Cell proliferation was determined by the mixing of DT40 cells with a fixed number of CaliBrite beads (Becton Dickinson). The numbers of beads and cells were counted simultaneously by FACS Scan flow cytometry. Apoptosis was measured by staining with FITC-conjugated Annexin V (Sigma) according to the manufacturer's protocol, followed by FACS analysis.

### Cell-Cycle Analyses

Cells were grown with or without drug and where indicated pulsed with 10 μM BrdU for 10 min before being harvested by centrifugation. Cells were fixed with 70% ethanol overnight at −20°C. After fixation, DNA was denatured by 30 min incubation in 2 M HCl and 0.5% Triton X-100. Samples were subsequently neutralized with 0.1 M Na_2_B_4_O_7_ (pH 8.5) before being stained with fluorescein isothiocyanate (FITC)-conjugated anti-BrdU (BD Biosciences). Cells were resuspended in phosphate-buffered saline (PBS) containing 5 mg/ml propidium iodide (Sigma) and analyzed by FACS Scan flow cytometry. For synchronization, cells were arrested (in G2/M) by treatment with nocodazole (25 ng/ml) for 8 hr, followed by washes and release.

### Western Blot

Cells were lysed in radioimmunoprecipitation assay (RIPA) buffer (1% NP40, 0.5% Na deoxycholate, 0.1% sodium dodecyl sulfate [SDS] and protease inhibitor cocktail in PBS) by syringing eight times through a 25 G needle. Lysates were cleared by centrifugation. For the analysis of FANCD2, samples were separated on 8% polyacrylamide Tris-Glycine gels by SDS-PAGE (polyacrylamide gel electrophoresis). Rabbit anti-FANCD2 polyclonal and goat anti-rabbit IgG conjugated to horseradish peroxidase (HRP) (Southern Biotech) were used as the primary and secondary antibody, respectively. For the analysis of EGFP-USP1 and PCNA, samples were separated by SDS-PAGE (10% Bis-Tris gels [Invitrogen]). For the detection of EGFP, rabbit anti-GFP antibody (Abcam) and goat anti-rabbit IgG conjugated to HRP (Southern Biotech) were used as the primary and secondary antibody, respectively. So that PCNA could be detected, mouse anti-PCNA (Abcam) and rabbit anti-mouse IgG conjugated to HRP (Dako) were used as the primary and secondary antibody, respectively.

### DNA Damage Sensitivity Assays

Mutagen sensitivity was investigated with the colony survival assay as described (Niedzwiedz et al., 2004). UV light at 254 nm was delivered with a Stratalinker (Stratagene). X-rays were delivered with a RX-650 radiation source (Faxitron). Cisplatin and mitomycin C were obtained from Sigma.

### IgM-Positive to -Negative Reversion Fluctuation Assay and V Gene Sequence Analysis

A comprehensive and detailed description of the method is described previously (Sale et al., 2001; Simpson and Sale, 2003). In brief, the MoFlo FACS sorter was used for the sorting of single IgM+ cells into 96-well plates. For each data set (each cell line compared), a minimum of 24 clones was expanded for a further month. After this time frame, the clones were stained with FITC-conjugated goat anti-chicken IgM on ice and analyzed on a FACS Scan cytometer for sIgM expression. For the sequencing of V genes from Ig-loss variants, the Ig-negative population from each of six independent clones was enriched by FACS sorting with a MoFlo cell sorter (Cytomation). Genomic DNA for each clone was prepared as described and the V lambda gene was amplified with primers CVL5 (5′-CAGGAGCTCGGCTCTGTCCCATTGCTGCGCGG-3′) and CVLR3 (5′-GCGCAAGCTTCCCCAGCCTGCCGCCAAGTCCCAAG-3′) for 30 cycles of polymerase chain reaction (PCR) amplification with Pfu turbo (Stratagene). Products were cloned into Blue Script and sequenced. A simple algorithm was used for the assignment of alteration in sequence as a gene conversion, as ambiguous, or as an untemplated mutation, as previously published (Sale et al., 2001). Founder or repeat sequences were scored once, and duplicates were removed from the analysis.

### Mutagenesis of USP1 cDNA

The chicken USP1 complementary DNA (cDNA) fused to the EGFP coding sequence was a kind gift of Dr. M. Takata. The C92S mutation in USP1 was generated by the Quikchange mutagenesis method with the following primers: forward (5′-CTTGGCAACACTAGTTACCTTAACAGCGTTCTTCAG-3′) and reverse (5′-AACGCTGTTAAGGTAACTAGTGTTGCCAAGATTATTCAA-3′). The G680A-G681A mutation (GG-AA) in USP1 was generated by the Quikchange mutagenesis method with the following primers: forward (5′-ATGGAAGCTGTGGGACTGCTAGCAGCACAGAAGAGCAAGAGCAAGTCT-3′) and reverse (5′-GCTCTTGCTCTTCTGTGCTGCTAGCAGTCCCACAGCTTCCATGT-3′).

## Supplemental Data

Document S1. Three Figures

## References

[bib1] Arakawa H., Moldovan G.L., Saribasak H., Saribasak N.N., Jentsch S., Buerstedde J.M. (2006). A role for PCNA ubiquitination in immunoglobulin hypermutation. PLoS Biol..

[bib2] Buerstedde J.M., Reynaud C.A., Humphries E.H., Olson W., Ewert D.L., Weill J.C. (1990). Light chain gene conversion continues at high rate in an ALV-induced cell line. EMBO J..

[bib3] Bienko M., Green C.M., Crosetto N., Rudolf F., Zapart G., Coull B., Kannouche P., Wider G., Peter M., Lehmann A.R. (2005). Ubiquitin-binding domains in Y-family polymerases regulate translesion synthesis. Science.

[bib4] Di Noia J., Neuberger M.S. (2002). Altering the pathway of immunoglobulin hypermutation by inhibiting uracil-DNA glycosylase. Nature.

[bib5] Di Noia J.M., Rada C., Neuberger M.S. (2006). SMUG1 is able to excise uracil from immunoglobulin genes: Insight into mutation versus repair. EMBO J..

[bib6] Friedberg E.C. (2006). Reversible monoubiquitination of PCNA: A novel slant on regulating translesion DNA synthesis. Mol. Cell.

[bib7] Garcia-Higuera I., Taniguchi T., Ganesan S., Meyn M.S., Timmers C., Hejna J., Grompe M., D'Andrea A.D. (2001). Interaction of the Fanconi anemia proteins and BRCA1 in a common pathway. Mol. Cell.

[bib8] Garg P., Burgers P.M. (2005). Ubiquitinated proliferating cell nuclear antigen activates translesion DNA polymerases {eta} and REV1. Proc. Natl. Acad. Sci. USA.

[bib9] Haracska L., Unk I., Prakash L., Prakash S. (2006). Ubiquitylation of yeast proliferating cell nuclear antigen and its implications for translesion DNA synthesis. Proc. Natl. Acad. Sci. USA.

[bib10] Hoege C., Pfander B., Moldovan G.L., Pyrowolakis G., Jentsch S. (2002). RAD6-dependent DNA repair is linked to modification of PCNA by ubiquitin and SUMO. Nature.

[bib11] Huang T.T., D'Andrea A.D. (2006). Regulation of DNA repair by ubiquitylation. Nat. Rev. Mol. Cell Biol..

[bib12] Huang T.T., Nijman S.M., Mirchandani K.D., Galardy P.J., Cohn M.A., Haas W., Gygi S.P., Ploegh H.L., Bernards R., D'Andrea A.D. (2006). Regulation of monoubiquitinated PCNA by DUB autocleavage. Nat. Cell Biol..

[bib13] Kannouche P.L., Wing J., Lehmann A.R. (2004). Interaction of human DNA polymerase eta with monoubiquitinated PCNA: a possible mechanism for the polymerase switch in response to DNA damage. Mol. Cell.

[bib14] Kawamoto T., Araki K., Sonoda E., Yamashita Y.M., Harada K., Kikuchi K., Masutani C., Hanaoka F., Nozaki K., Hashimoto N., Takeda S. (2005). Dual roles for DNA polymerase eta in homologous DNA recombination and translesion DNA synthesis. Mol. Cell.

[bib15] Meetei A.R., Medhurst A.L., Ling C., Xue Y., Singh T.R., Bier P., Steltenpool J., Stone S., Dokal I., Mathew C.G. (2005). A human ortholog of archaeal DNA repair protein Hef is defective in Fanconi anemia complementation group M. Nat. Genet..

[bib16] Montes de Oca R., Andreassen P.R., Margossian S.P., Gregory R.C., Taniguchi T., Wang X., Houghtaling S., Grompe M., D'Andrea A.D. (2005). Regulated interaction of the Fanconi anemia protein, FANCD2, with chromatin. Blood.

[bib17] Mosedale G., Niedzwiedz W., Alpi A., Perrina F., Pereira-Leal J.B., Johnson M., Langevin F., Pace P., Patel K.J. (2005). The vertebrate Hef ortholog is a component of the Fanconi anemia tumor-suppressor pathway. Nat. Struct. Mol. Biol..

[bib18] Niedzwiedz W., Mosedale G., Johnson M., Ong C.Y., Pace P., Patel K.J. (2004). The Fanconi anaemia gene FANCC promotes homologous recombination and error-prone DNA repair. Mol. Cell.

[bib19] Nijman S.M., Huang T.T., Dirac A.M., Brummelkamp T.R., Kerkhoven R.M., D'Andrea A.D., Bernards R. (2005). The deubiquitinating enzyme USP1 regulates the Fanconi anemia pathway. Mol. Cell.

[bib20] Nijman S.M., Luna-Vargas M.P., Velds A., Brummelkamp T.R., Dirac A.M., Sixma T.K., Bernards R. (2005). A genomic and functional inventory of deubiquitinating enzymes. Cell.

[bib21] Ross A.L., Sale J.E. (2006). The catalytic activity of REV1 is employed during immunoglobulin gene diversification in DT40. Mol. Immunol..

[bib22] Sale J.E., Calandrini D.M., Takata M., Takeda S., Neuberger M.S. (2001). Ablation of XRCC2/3 transforms immunoglobulin V gene conversion into somatic hypermutation. Nature.

[bib23] Simpson L.J., Sale J.E. (2003). Rev1 is essential for DNA damage tolerance and non-templated immunoglobulin gene mutation in a vertebrate cell line. EMBO J..

[bib24] Simpson L.J., Ross A.L., Szuts D., Alviani C.A., Oestergaard V.H., Patel K.J., Sale J.E. (2006). RAD18-independent ubiquitination of proliferating-cell nuclear antigen in the avian cell line DT40. EMBO Rep..

[bib25] Smogorzewska A., Matsuoka S., Vinciguerra P., McDonald E.R., Hurov K.E., Luo J., Ballif B.A., Gygi S.P., Hofmann K., D'Andrea A.D., Elledge S.J. (2007). Identification of the FANCI protein, a monoubiquitinated FANCD2 paralog required for DNA repair. Cell.

[bib26] Taniguchi T., Garcia-Higuera I., Andreassen P.R., Gregory R.C., Grompe M., D'Andrea A.D. (2002). S-phase-specific interaction of the Fanconi anemia protein, FANCD2, with BRCA1 and RAD51. Blood.

[bib27] Ulrich H.D. (2006). Deubiquitinating PCNA: A downside to DNA damage tolerance. Nat. Cell Biol..

[bib28] Zhou B.B., Elledge S.J. (2000). The DNA damage response: Putting checkpoints in perspective. Nature.

