# Supplementary material for: Deubiquitination of FANCD2 Is Required for DNA Crosslink Repair
Source: Mol Cell. 2007 Dec 14;28(5):798–809. doi: 10.1016/j.molcel.2007.09.020 (PMC2148256; doi:10.1016/j.molcel.2007.09.020)
Supplement: Document S1. Three Figures [file mmc1.pdf]

Molecular Cell, Volume *28*

Supplemental Data

Deubiquitination of FANCD2 Is Required for DNA Crosslink Repair

Vibe H. Oestergaard, Frederic Langevin, Hendrik J. Kuiken, Paul Pace, Wojciech Niedzwiedz, Laura J. Simpson, Mioko Ohzeki, Minoru Takata, Julian E. Sale, and Ketan J. Patel

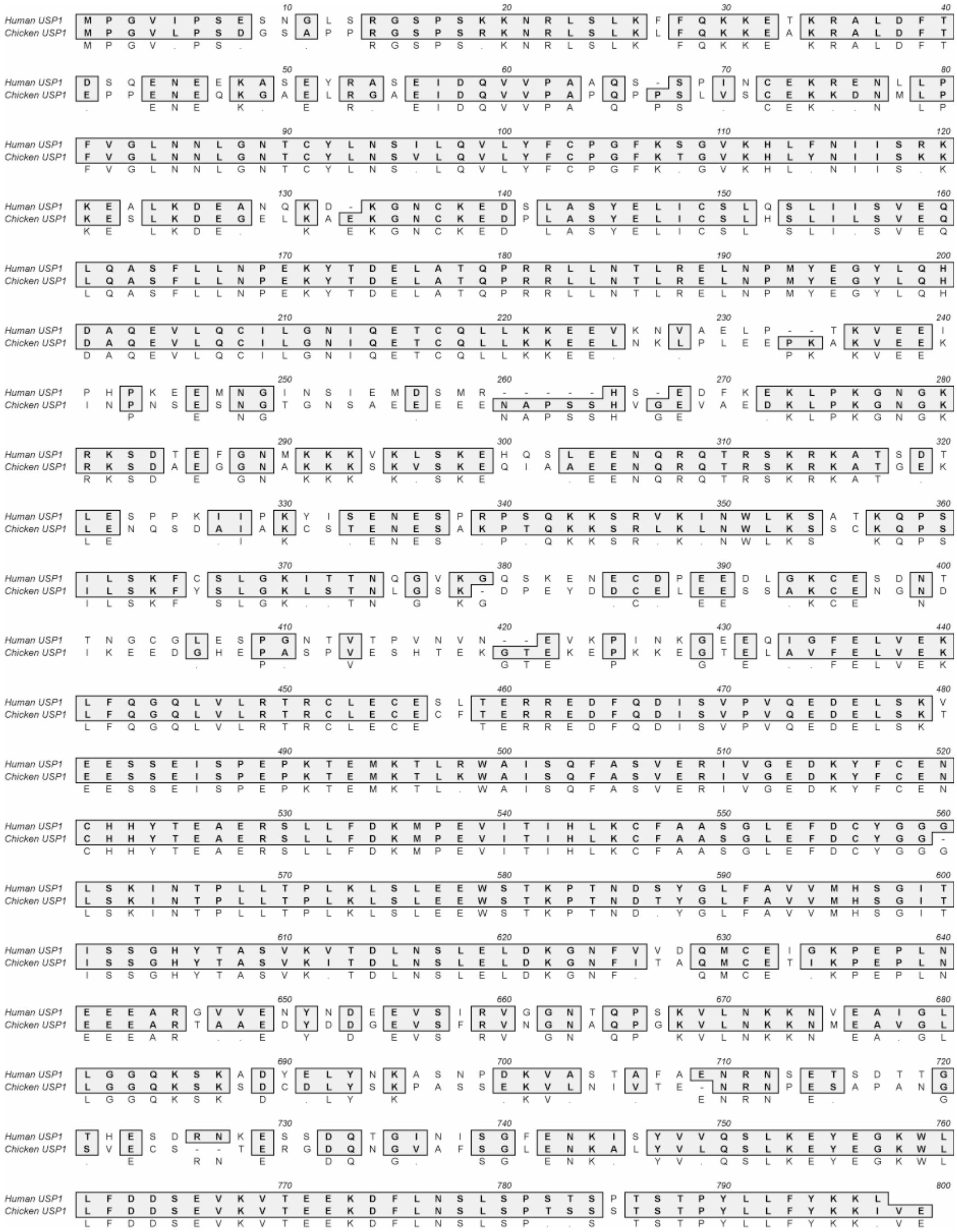

Figure S1. Alignment of the Human and Chicken USP1 Protein Sequences
The sequences share 73% identity.

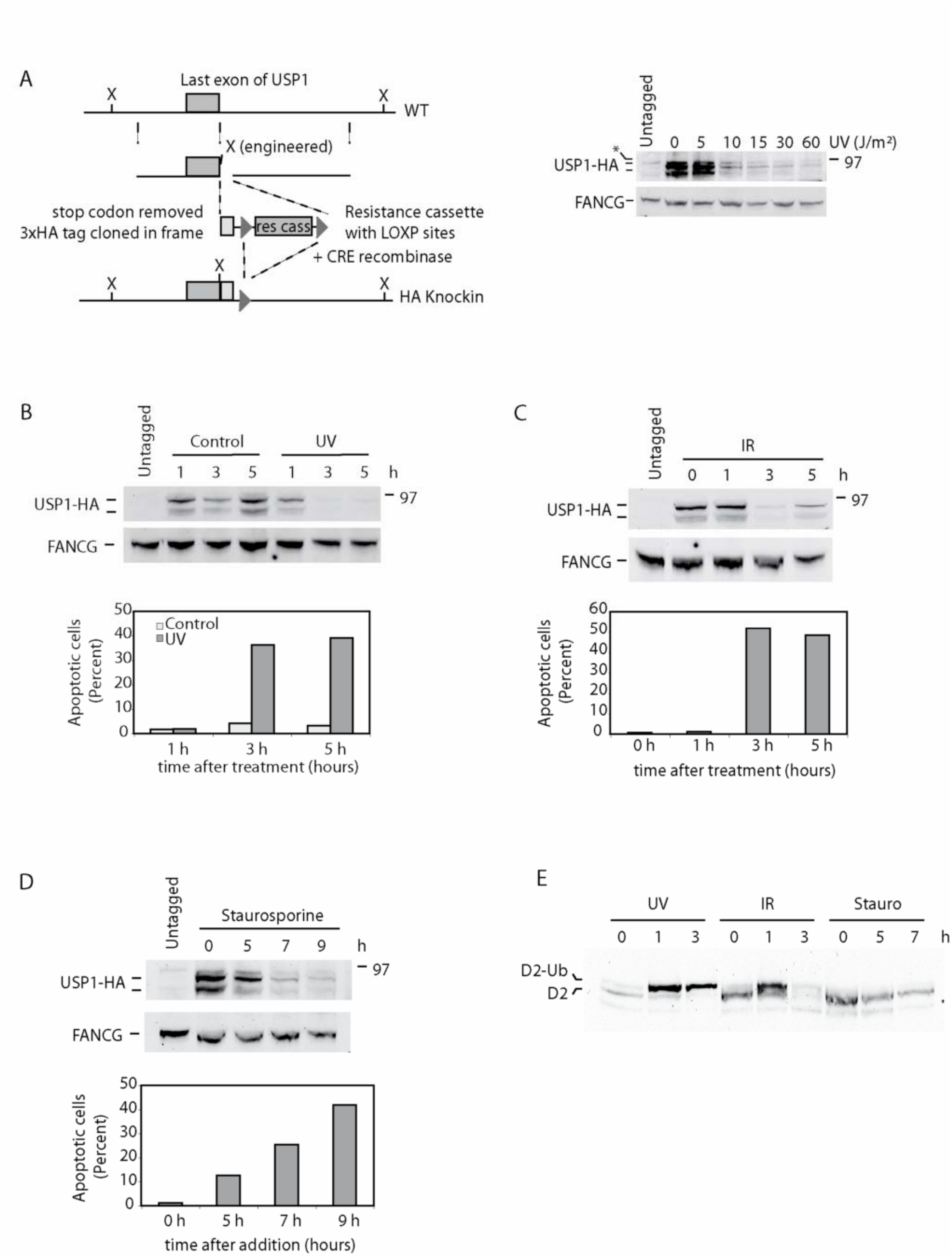

Figure S2. USP1 Degradation Monitored in USP1 *In Situ* HA-Tagged Cells
(A) Left - map of the *USP1* locus and strategy for the generation of the *in situ* HA-tag into the last exon of *USP1*. After recycling the drug resistant cassette the locus expresses USP1-3xHA tag. Right - Western blot to detect USP1-HA after exposing cells to different doses of UV light. The asterisk denotes a cross reacting band. For unknown reasons the HA antibody recognises two specific bands for the USP1-HA tagged cell line. The lower band, however, is not a USP1 self cleavage product. The C-terminal HA-tagged self cleavage product would be around 15 kDa. Western blot against FANCG was used as a loading control.

(B) Top –HA and FANCG Western blots from cells exposed to UV light (30 J/m^2) recovered for 1, 3 and 5 hours. Below – column chart showing the percentage of apoptotic cells detected by uptake of Annexin V and FACS analysis at the same time points.
(C) Top - HA and FANCG Western blots from cells exposed to X-rays (20 GY). Below – graph plotting the percentage of apoptotic cells detected by uptake of Annexin V and FACS analysis at the same time points.
(D) Top – HA and FANCG Western blots from cells exposed to single dose of the apoptosis-inducing drug staurosporine. Samples removed at 5, 7 and 9 hours after exposure to drug. Graph showing percentage of apoptotic cells detected by uptake of Annexin V and FACS analysis at the same time points.
(E) FANCD2 Western blot performed on cells treated with UV, X-rays or staurosporine.

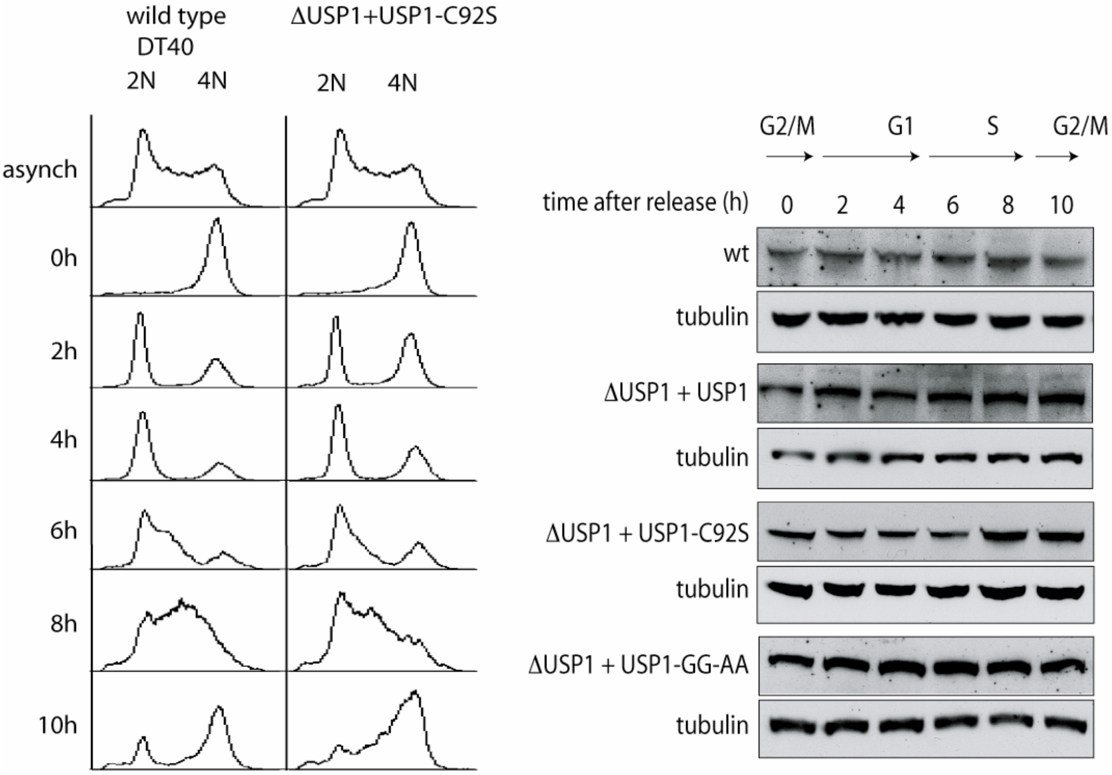

Figure S3. Following the Levels of USP1 Wild Type and Mutants throughout the Cell Cycle

Wild type DT40 and Δ*USP1* stably transfected with mutant or wild type USP1 cDNA were synchronised (in G2/M). Two hourly samples were analysed by FACS and immunoblotted with anti-USP1 and anti-tubulin (as a loading control) antibodies. USP1 and USP1-GG-AA transfected cells display identical synchrony to wild type cells as above (data not shown).
